# Dynamics and impact of footrot and climate on hoof horn length in 50 ewes from one farm over a period of 10 months

**DOI:** 10.1016/j.tvjl.2014.05.021

**Published:** 2014-09

**Authors:** Edward M. Smith, Olivia D.J. Green, Leonides A. Calvo-Bado, Luci A. Witcomb, Rosemary Grogono-Thomas, Claire L. Russell, Judith C. Brown, Graham F. Medley, Amy L. KilBride, Elizabeth M.H. Wellington, Laura E. Green

**Affiliations:** a School of Life Sciences, University of Warwick, Gibbet Hill Road, Coventry CV4 7AL, UK; b Department of Clinical Veterinary Sciences, University of Bristol, Langford House, Langford, Bristol BS40 5DU, UK

**Keywords:** Climate, Footrot, Hoof horn length, Longitudinal study, Sheep

## Abstract

Footrot, including interdigital dermatitis, is caused by *Dichelobacter nodosus* cause the majority of lameness in sheep in the UK. Lame sheep often have overgrown hoof horn but recent evidence has indicated that trimming overgrown hoof horn increases recovery time, and that routine foot trimming of the flock does not reduce the prevalence or incidence of lameness. The objectives of this study were to investigate the temporal associations between hoof horn length, footrot and climate. Fifty multiparous ewes were monitored for 10 months. On eight occasions hoof horn length, foot lesions and body condition were recorded. At the first examination, ewes were assigned to one of two treatment groups. All ewes that became lame with footrot were treated at one time point per week, either by trimming hoof horn and applying a topical antibiotic spray or with parenteral antibiotic and topical antibiotic spray.

Hoof horn length in ewes at pasture varied over the year and was associated with temperature and rainfall. New cases of footrot occurred all year round and were associated with prior prevalence of footrot in the flock and prior temperature and rainfall. Overgrown hoof horn did not precede lameness but occurred once the sheep were lame. One year of prompt treatment of footrot reduced the range in hoof horn length in the sheep in both treatment groups. At the end of the study the hoof lengths of ewes in both groups were not significantly different. On this farm, hoof horn length was self-regulating in both non-lame and treated lame sheep whether trimming was part of the treatment or not and there would have been no benefit from routine foot trimming of this flock.

## Introduction

Lameness in sheep causes pain (Ley et al., 1989) and reduces productivity (Wassink et al., 2010b). Two previous studies from random samples of British farmers estimated that the prevalence of lameness in 1997 and 2004 was approximately 10% (Grogono-Thomas, Johnston, 1997, Kaler, Green, 2009). Using a similar study design in 2011, the median lameness was 5% (King, 2013), suggesting a downward trend in prevalence of lameness in the UK over the last 5–10 years.

Footrot (FR), including interdigital dermatitis (ID), is responsible for approximately 90% of all lameness in sheep in Great Britain (GB) (Kaler, Green, 2008, Kaler, Green, 2009) and both presentations of disease are caused by the bacterium *Dichelobacter nodosus* (Beveridge, 1941, Moore et al, 2005, Raadsma, Egerton, 2013). The occurrence and spread of FR in countries is influenced by local climatic conditions (Green and George, 2008). When the seasonal climate is conducive to spread of *D. nodosus*, epidemics of FR occur; when the seasonal climate is dry, there is no spread of the bacterium and the prevalence of FR falls to zero. In Australia, a mean daily temperature >10 °C and wet pasture are necessary for the development and spread of FR (Graham and Egerton, 1968). In contrast, Ridler et al. (2009) reported that FR occurs throughout the year in GB, even when the mean daily temperature is <10 °C. This agrees with Green et al. (2007) who described a series of mini-epidemics of FR throughout the year in a flock of sheep in GB, related to the number of diseased sheep in previous weeks and flock management of FR.

Routine foot trimming (where farmers trim the hoof horn of all sheep whether they have signs of lameness or not) is a flock management tool for lameness control that has been practised for many years in GB (Morgan, 1987, Winter, 2004). Those sheep farmers who routinely foot trim their flock do so once, twice or more times per year (Wassink et al., 2003), with farmers stating that they use this procedure to both treat and control FR (Wassink et al, 2005, Wassink et al, 2010b). However, an association between the number of routine foot trimming events per year and a higher prevalence of flock lameness has been reported (Wassink et al, 2003, Kaler, Green, 2009). One explanation for this is that trimming hoof horn spreads *D. nodosus* between sheep through close contact and dirty equipment and so directly increases the incidence of lameness, especially if sensitive tissue is exposed when hoof horn is trimmed. It is also possible that the statistical association between prevalence of lameness and routine foot trimming is an indirect association i.e. routine foot trimming is correlated with other management procedures that increase the prevalence of lameness but is itself not causally associated with increased lameness.

Irrespective of this association, routine foot trimming does not reduce the prevalence of lameness in subsequent weeks (Green et al., 2007) or in the subsequent year (Kaler and Green, 2009), suggesting that even if trimming is conducted in response to high levels of lameness it is not effective. Despite the finding that lame sheep are likely to have overgrown hoof horn, Kaler et al. (2010) reported that trimming the hoof horn as part of the treatment for FR actually delayed recovery compared with no foot trimming (all sheep were also treated with parenteral and topical antibiotics): 75% versus <5% of sheep, respectively, were still lame 10 days after treatment.

Approximately 25% of English farmers do not practise routine foot trimming (Kaler and Green, 2009) and anecdotally report that they have had fewer lame sheep or no difference in lameness as a result of stopping routine foot trimming. Nevertheless, some farmers and specialists are adamant that routine foot trimming is an essential activity. Their reasons focus on the need to remove overgrown horn: (1) to ensure that soil does not collect and compress in the overgrown wall horn and predispose sheep to FR or a bruised sole (if the soil dries and acts like a small stone); (2) to enable sheep to walk unimpeded by long toes or by wall horn curling under the sole; and (3) to ensure there is no excess horn that could break off and damage the foot, e.g. the toe breaking off and exposing the sensitive laminae of the foot (King, 2013).

Hoof horn protects the sensitive internal structures of the foot and allows animals to walk on hard and irregular surfaces without pain. Very little is known about the nature of sheep hoof horn but several studies on hoof horn in dairy cows provide some basic principles of its likely nature. In dairy cows, horn grows continuously; it is moderated by the environment e.g. it absorbs moisture and so is softer and more flexible in animals kept in wet conditions (Gregory et al., 2006) and harder and more brittle when animals are in dry conditions. In addition, the growth rate and wear of hoof horn vary with the hardness of the floor surface (Vokey et al., 2001). If wear is slower than growth, then hoof horn grows distal to the sole of the foot. Thus, sheep hoof horn is affected by floor type; in 21 healthy yearling ewes at pasture, hoof horn growth rate was 3.3 mm/month (Shelton et al., 2012) while in five lambs and five ewes on concrete, hoof horn growth rates were 13.2 mm/month and 8.7 mm/month, respectively (Dekker et al., 2005). It is also affected by temperature; Wheeler et al. (1972) reported growth rates of 0.7–6.0 mm/month in four groups of 8–21 sheep, and that sheep kept in low ambient temperatures had a slower hoof horn growth rate.

Investigation of the dynamics of sheep hoof horn length over time and its relationship to FR is therefore timely. In the current study we investigated the temporal patterns of hoof horn length and horn length associations with FR, therapeutic foot trimming and temperature and rainfall, and evaluated how these results can inform the likely usefulness of routine foot trimming.

## Materials and methods

### Ethical approval

This study was granted approval by the University of Bristol Ethics Committee, UIN number UB/09/020.

### Flock selection

A flock of 99 Mule and Suffolk-cross ewes was selected for study based on a history of lameness caused by FR, and likely compliance of the farm staff with study protocols. The flock was mated to five Texel and Suffolk rams in November 2010. Barren ewes were separated from the main flock in early February 2011, and pregnant ewes were offered supplementary feed after this date. Pregnant ewes were housed for lambing in late March, and lambing occurred over a 3-week period in March and April 2011. Ewes were housed for between 3 and 21 days. Ewes and lambs were turned out to pasture approximately 24 h postpartum. The flock was maintained as a single population of ewes with lambs at foot until the lambs were weaned in August at approximately 12 weeks of age. Orphan lambs were kept separate from the main flock, and barren ewes were re-introduced into the flock in May 2011.

### Study design

The study ran from October 2010 to August 2011. Fifty ewes were selected for detailed study. Ewes were assigned to one of two groups (Inject or Trim; to be described later) by stratified random sampling on body condition, using a scale from 0 to 5 (Defra, 1997), age (dentition), ID and FR lesion scores and foot conformation (integrity) (Foddai et al., 2012).

Ewes were examined during four non-consecutive 4-week periods (October/November 2010; January 2011; May 2011; August 2011). Foot lesions were recorded each week, the body condition score (BCS) of each ewe was recorded at week 1, and foot conformation and hoof horn length were recorded in weeks 1 and 4. Hoof length was estimated visually at the mid-point of the abaxial wall and point of the toe of each digit, by one researcher. This was scored as: 0, level with the sole; −0.5, −1 to −5 mm; −1.0 = −6 to −10 mm etc. for wall or toe horn length proximal to the sole, and; 0.5, 1 to 5 mm, 1.0, 6 to 10 mm etc. for wall and toe horn length distal to the sole.

In addition to the 4-week periods of routine observation, the locomotion of the flock was scored each week by a trained researcher (EMS), using a validated 0–6 scoring scheme (Kaler et al., 2009). When a ewe had a locomotion score (LS) >2 (minimum lameness was holding a foot up when standing, but weight bearing when walking) she was turned and her body condition, foot conformation, foot lesions (Foddai et al., 2012) and hoof horn length were recorded.

FR was defined as ID with or without separation of the hoof horn from the sensitive tissue beneath together with a characteristic smell. Ewes in Inject with ‘FR and locomotion score >2’ (FR >2) were treated with 2000 mg oxytetracycline (10 mL Oxytetrin 20 LA [200 mg/mL]; Intervet UK) by intramuscular injection and topical application of oxytetracycline (Engemycin Spray; Intervet UK) to all feet. Ewes in Trim with FR >2 were treated by trimming all feet of the lame ewe to remove areas of overgrowth and under-running horn and topical application of oxytetracycline to all feet.

All treated ewes were monitored for 2 weeks and, with the exception of the first 8 weeks of pregnancy, were locomotion scored and their feet examined each week. If a ewe had FR >2 for 2 consecutive weeks, treatment was repeated and the ewe monitored for a further 2 weeks. Ewes in Trim that had FR >2 for 4 weeks consecutively were given 2000 mg oxytetracycline and their feet were sprayed with topical oxytetracycline.

### Data recording

All data were recorded on custom-designed paper recording sheets (Supplementary File 1 in the online version at doi:10.1016/j.tvjl.2014.05.021). Each week the data were loaded into an Access database (Microsoft) located on a secure server at The University of Warwick. Weather data for the farm site were downloaded each month from a private weather station website^1^ and stored in an Excel database.

### Data analysis

Correlations between the wall and toe horn lengths were investigated within digit, foot and ewe. The patterns of wall and toe lengths were plotted over time. Differences in the variation in measures at the start and end of the study were assessed using F-tests of variance. The impact of 2-week mean (minimum, mean and maximum) daily temperature in the 2 weeks preceding the observation, 2-week mean daily rainfall, age, body condition, locomotion score, FR and treatment for FR on hoof horn length were examined using a mixed effect continuous outcome model that took the form:

$$\text{Hoof}\;\text{horn}\;{\text{length}}_{ijk}=\beta_{0}+\sum \beta x_{ijk}+\sum \beta x_{jk}+\sum \beta x_{k}+f_{k}+v_{jk}+u_{ijk}$$

where hoof horn length*_ijk_* = the length of the hoof horn on occasion *ijk*, where *i* is observation, *j* is foot and *k* is ewe. *β*_0_ = intercept, *β*x is a vector of fixed effects varying at level 1 (*ijk*), level 2 (*jk*), and level 3 (*k*), *f_k_, v_jk_* and *u_ijk_* are the level 3, 2 and 1 residual variances, respectively.

The associations between the occurrence of FR >2 and temperature, rainfall, force of infection (number of sheep lame with FR or ID in previous weeks), length of hoof horn and ewe body condition in the preceding and current weeks were explored for all 50 ewes. A mixed effect logistic binomial regression model was built that took the form:

$$\text{Logit}\;{\left(\text{FR}\;>\;2\right)}_{ijk}=\beta_{0}+\sum \beta x_{ijk}+\sum \beta x_{jk}+\sum \beta x_{k}+f_{k}+v_{jk}$$

where (FR >2)*_ijk_* = a binary variable for whether a FR-affected foot came from a sheep with locomotion score >2 or not, with a logit link with *β*_0_ = intercept, *β*x is a vector of fixed effects varying at level 1 (*ijk*), level 2 (*jk*), and level 3 (*k*) where *i* is observations, *j* is foot and *k* is ewe, *f_k_*, and *v_jk_* are the level 3 and 2 residual variances respectively, level one was constrained to a binomial distribution. For this model, hoof horn length was categorised into a binary variable overgrown 0/1 where 0 = horn proximal to, or level with, the sole and 1 = horn distal to the sole. Model fit was tested using the Hosmer Lemeshow test.

## Results

### Hoof horn length and factors associated with change in length

There was no significant difference in hoof horn length by original allocation to group, BCS or age (Supplementary File 2 in the online version at doi:10.1016/j.tvjl.2014.05.021). Four ewes were lost to follow up during the trial. Over the 46 weeks of the study, there were 9920 foot length observations (Table 1). There were eight routine examinations, giving 6224 routine observations (four observations per foot) and 3696 non-routine observations of hoof horn length.

**Table 1:** Distribution of locomotion scores and hoof horn length observations between the Inject and Trim groups in 50 ewes from one flock in Somerset, England.

Locomotion score	Sheep observations		Hoof length observations	
	Inject group (*n*)	Trim group (*n*)	Inject group (*n*)	Trim group (*n*)
NR	25	25	400	400
0	937	1010	3248	3376
1	18	17	32	48
2	69	25	400	112
3	59	55	928	864
4	6	1	96	16
Total	1114	1133	5104	4816

NR, not recorded.

**Fig. 1:**
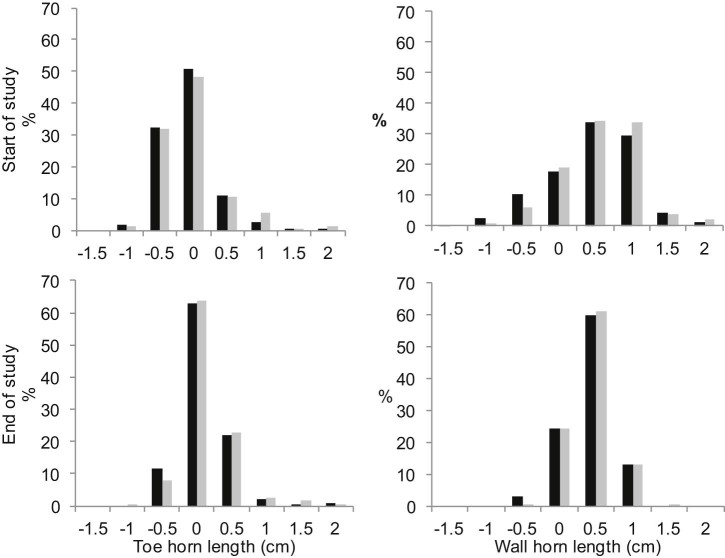
Percent of observations with toe and wall length distal (+ values) and proximal (–values) to the sole (0) in centimetres at the start (October 2010) and end (August 2011) of a study of 50 sheep, 200 feet, from one farm. Hoof lengths > 2.0 cm are included in the 2.0 cm category for display purposes. Black bars: Inject group (treatment for footrot by injection of oxytetracycline). Grey bars: Trim group (treatment for footrot by foot trimming).

**Fig. 2:**
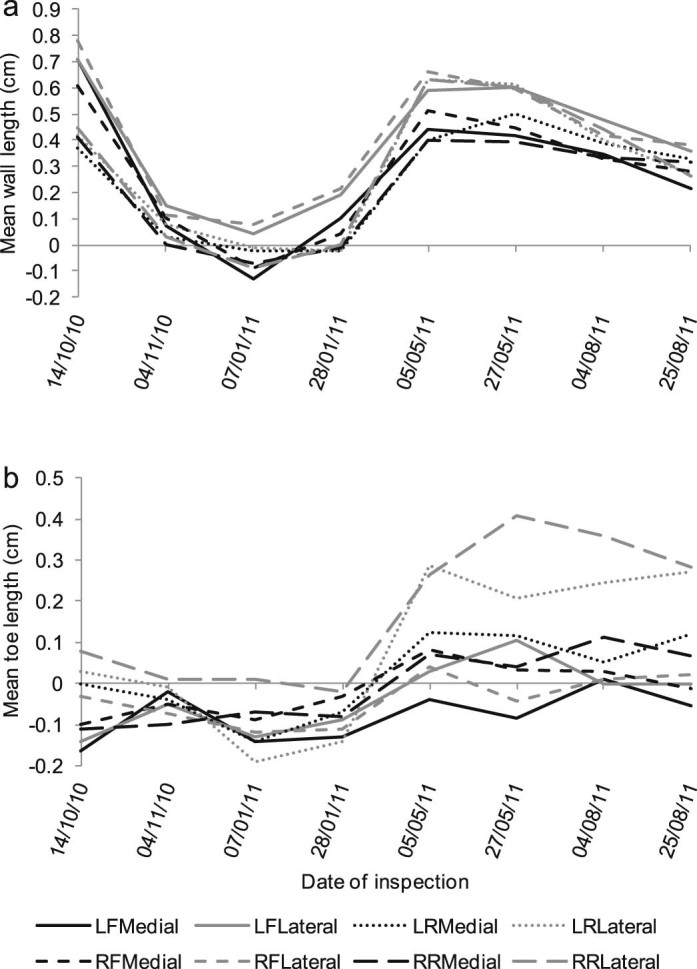
Change in (a) wall and (b) toe length by medial and lateral digits of 50 ewes observed on eight occasions from October 2010 to August 2011, length zero is level with the sole. LF, left fore; LR, left rear; RF, right fore; RR, right rear; Medial, medial digit; Lateral, lateral digit. Note: horizontal axes are not to scale.

Wall horn length varied more than toe hoof horn length over time (Fig. 1, Fig. 2). Mean observed wall hoof horn length was −0.1 to +0.8 cm distal to the sole while mean observed toe length extended −0.2 to +0.4 cm distal to the sole. There were clear patterns in hoof horn length over time. Wall horn length shortened from October 2010 to January 2011, and in January the wall and toe horn were approximately level with the sole. Horn lengthened from January to May, and shortened again from May to August 2011 (Fig. 2). The variability in wall and toe horn length reduced from the start to the end of the study in both the Trim and Inject groups (F-test of variance: *P* < 0.05 for all groups; Fig. 1).

The factors associated with hoof horn length are summarised in Table 2. The mean hoof horn lengths of the medial and lateral digit walls and lateral digit toe were significantly longer than the mean hoof horn length of the medial digit toe. Sheep with BCS = 1–2.5 had significantly longer hoof horn length than those with BCS = 5, but sheep with BCS = 3–4.5 did not have significantly different hoof horn length from sheep with BCS = 5. An increased 2-week rolling mean minimum daily temperature in the 2 weeks preceding the observation week was associated with shorter hoof horn length; and higher 2-week rolling mean daily rainfall in the preceding 2 weeks was significantly associated with longer hoof horn length (Table 2).

**Table 2:** Mixed effects linear regression model of factors associated with hoof horn length in 50 ewes, 784 feet and 8520 observations from one farm in Somerset, England, 2010–2011.

Fixed part	Mean distance from sole (cm)	SE	Lower 95% CI	Upper 95% CI
Intercept	0.199	0.126	−0.048	0.446
Trim group	Baseline			
Inject group	−0.002	0.031	−0.063	0.059
Hoof horn length, inner toe	Baseline			
Inner wall	0.233	0.019	0.196	0.270
Outer toe	0.076	0.019	0.039	0.113
Outer wall	0.319	0.019	0.282	0.356
Body condition score 5	Baseline			
Body condition score 1–1.5	0.189	0.045	0.101	0.277
Body condition score 2–2.5	0.057	0.017	0.024	0.090
Body condition score 3–3.5	0.013	0.012	−0.011	0.037
Body condition score 4–4.5	0.003	0.011	−0.019	0.025
Age < 4years	Baseline			
Age 4 years	−0.068	0.110	−0.284	0.148
Age > 4 years	−0.026	0.103	−0.228	0.176
Mean 2-weekly daily rainfall	0.018	0.004	0.010	0.026
Mean minimum 2-week temperature	−0.020	0.005	−0.030	−0.010
Non-lame feet	Baseline			
Foot locomotion score 1 at *t*-1	−0.013	0.075	−0.160	0.134
Foot locomotion score 2 at *t*-1	0.103	0.032	0.040	0.166
Foot locomotion score 3 at *t*-1	0.007	0.019	−0.030	0.044
Foot locomotion score 4 at *t*-1	0.096	0.069	−0.039	0.231
No treatment at *t*-1	Baseline			
Injected FR >2 at *t*-1	0.054	0.015	0.025	0.083
Trimmed FR >2 at *t*-1	−0.139	0.016	−0.170	−0.108
No treatment at *t*-2	Baseline			
Injected FR >2 at *t*-2	0.045	0.014	0.018	0.072
Trimmed FR >2 at *t*-2	−0.107	0.015	−0.136	−0.078
October 2010	Baseline			
November 2010	−0.275	0.031	−0.336	−0.214
December 2010	−0.469	0.067	−0.600	−0.338
January 2011	−0.489	0.061	−0.609	−0.369
February 2011	−0.375	0.054	−0.481	−0.269
March 2011	−0.158	0.046	−0.248	−0.068
April 2011	−0.223	0.050	−0.321	−0.125
May 2011	0.047	0.033	−0.018	0.112
June 2011	0.044	0.025	−0.005	0.093
July 2011	0.072	0.026	0.021	0.123
August 2011	0.071	0.020	0.032	0.110
Random terms	Variance	S.D.		
Level: Sheep	0.008	0.002		
Level: Foot	0.025	0.002		
Level: Week	0.104	0.002		

SE, standard error of the mean; 95% CI, 95% confidence interval; +, distal to sole; −, proximal to sole.

The maximum and minimum temperatures were highly correlated and so only 2-week rolling mean minimum temperature was left in the final model. A locomotion score of 2 (nodding of the head and a shortened stride) 1 week before (*t*-1) observation *i* at time *t* was associated with significantly longer hoof horn length in the affected foot. All sheep with locomotion score >2 had longer hoof horn before treatment. Compared with untreated sheep, sheep in the Trim group 1 and 2 weeks after treatment had significantly shorter hoof horn length while sheep in the Inject group had significantly longer hoof horn length (Table 2). At the end of the study there was no significant difference in the length of hoof horn between sheep in the Trim or Inject groups (Fig. 1). Visual inspection of the residuals indicated that the model fit was good (Supplementary File 3 in the online version at doi:10.1016/j.tvjl.2014.05.021).

### Factors associated with development of footrot with locomotion score >2

**Fig. 3:**
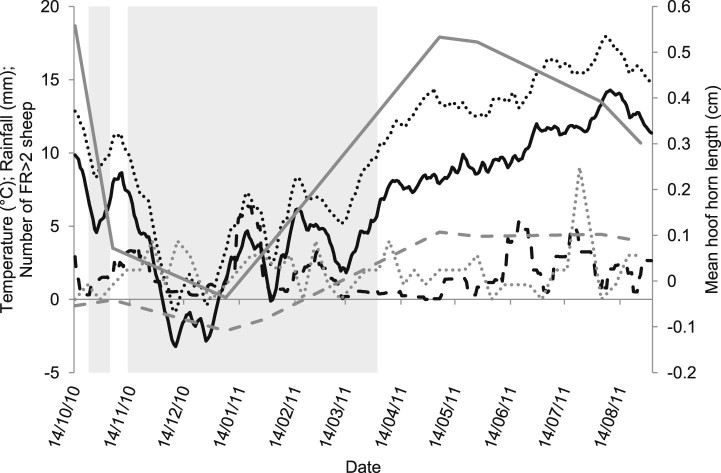
Mean 2-weekly temperature in degree Celsius (dotted black line), minimum temperature in degree Celsius (solid black line) and rainfall in millimetres (dashed black line) for the location of the study flock during the study period. Mean wall (solid grey line) and toe (dashed grey line) length is displayed and the number of sheep with footrot with locomotion score >2 (dotted grey line) in 50 ewes from one flock from October 2010 to August 2011. The shaded regions are the periods of the year when the mean 2-weekly temperature was <10 °C.

There were 153 treatments of ewes with FR and LS >2 (FR >2). There was no significant difference in the number of treatments by group (Table 3). Sheep were more likely to develop FR >2 as the number of sheep with FR in the preceding week increased and as the number of sheep with ID in the preceding 8–14 days decreased. There was a higher risk of FR >2 in April when ewes were housed for some of the month and also lambs were running with ewes. FR >2 was associated with 2-week rolling mean daily rainfall 1–2 weeks previously and 2-week rolling mean daily minimum temperature (Fig. 3; Table 3).

**Table 3:** Mixed effect binomial regression model of factors associated with development of footrot with locomotion score >2, at time *t*, in 50 ewes from one farm in Somerset, England 2010–2011.

Variables	Odds ratio	Lower 95% CI	Upper 95% CI
Predictors of FR >2 at time *t*-1			
Total number cases of footrot at *t*-1	1.08	1.03	1.14
Total number cases of ID at *t*-2	0.84	0.76	0.93
Mean minimum previous 2-week temperature	0.88	0.79	0.99
Mean minimum previous 2-week temperature squared	1.02	1.01	1.04
Mean previous 2-week daily rainfall at *t*	1.33	1.15	1.53
Mean previous 2-week daily rainfall at *t*-1	1.13	0.99	1.29
Group Inject versus Trim	0.80	0.45	1.44
October 2010	Baseline		
November 2010	1.53	0.65	3.57
December 2010	1.29	0.48	3.49
January 2011	1.26	0.50	3.17
February2011	1.89	0.94	3.84
March 2011	1.59	0.73	3.46
April 2011	3.22	1.57	6.60
May 2011	1.54	0.74	3.21
June 2011	0.67	0.30	1.46
July 2011	0.55	0.17	1.77
August 2011	0.22	0.05	1.02
Random effects	Variance	S.D.	
Level: Sheep	1.028	0.225	
Level: Foot	0.000	0.000	
Further associations with FR >2 when at time *t*, added to the model above			
*(small changes to fixed and random coefficients at t*-*1 not presented)*			
Hoof horn distal to sole – no	Baseline		
Hoof horn distal to sole – yes	3.26	2.34	4.54
Body condition score 3–3.5	Baseline		
Body condition score 1–1.5	2.83	1.32	6.07
Body condition score 2–2.5	0.73	0.49	1.08
Body condition score 4–4.5	1.08	0.76	1.53
Body condition score 5	0.43	0.26	0.73

95% CI, 95% confidence interval.

There was no association between BCS or hoof horn length and FR >2 in the week preceding its development. However, once the ewes were lame, they were significantly more likely than non-lame ewes to have a BCS = 1–1.5 and significantly less likely to have a BCS = 5 (Table 3). In addition, lame ewes were more likely to have longer hoof horn the week they were treated than ewes that were not treated. The model residual fit was good (Hosmer Lemeshow test: *P* > 0.05).

## Discussion

This is the first prospective study of the relationships between climate, hoof horn length and FR in ewes. One purpose of this research was to elucidate whether sheep with overgrown hoof horn were more likely to become lame with FR or vice versa.

Horn overgrowth was modelled at foot level and once feet were affected with FR horn was overgrown. Explanations for overgrown hoof horn on lame feet include: (1) not weight-bearing on an affected limb and so hoof horn is not worn away but continues to grow; (2) horn in diseased feet growing more rapidly; or (3) a combination of both. Once a sheep with FR was treated, the hoof horn on the affected limb reduced in length. Immediate trimming created an iatrogenic shortening of hoof horn length while ewes treated with an antibiotic injection and not trimmed did not have significantly longer hoof horn by the end of the study (Fig. 1). This is probably explained by previous findings where >95% of sheep with FR recovered within 10 days when following the Inject group treatment protocol (Kaler et al., 2010) and it is likely that normal weight bearing, and recovery from infection and lameness, led to no overgrown horn lengths by the end of the study. We conclude that overgrown hoof horn follows lameness caused by FR.

It has been suggested that animal weight affects pressure on the digits; in the current study thin sheep (BCS = 1 or 2) had longer hoof horn than ewes with BCS = 5. However, this relationship was only present once ewes were lame so the association between low BCS and long hoof horn might be due to lameness rather than weight of the sheep. BCS was also lower in lame sheep, but only once lame. We did not study BCS for sufficiently long after treatment to see whether and when ewes returned to higher BCS but Wassink et al. (2010a) found that BCS >2.5 was associated with prompt treatment.

The occurrence of FR >2 was driven by the force of infection, with increasing treatments following a week with increasing numbers of sheep with FR, and possibly with increasing stocking density (from April lambs were running with ewes). The significantly higher odds ratio for development of FR in April compared with other months might be explained by the altered environment of housing. Ewes were housed for 3–21 days in March/April during lambing and so experienced different underfoot conditions for this short period of time. There was also increased stocking density in pastures in April as lambs were born (Russell et al., 2013). The negative association between increasing ID 2 weeks previously and FR >2 might occur because sheep develop FR after ID (Kaler et al., 2011) and so as FR increases ID decreases (Green et al., 2007).

In the current study, horn length was measured in relation to the sole, not the rate of growth, which requires detailed marking on the hoof horn (Shelton et al., 2012). Measuring length in relation to sole rather than growth enables reporting of how the appearance of feet changed over the course of 10 months. From the measurements made in the present study, wall horn was typically longer than toe horn. It was interesting to note that hoof horn increased in length from winter to spring and shortened from mid-summer to autumn and paralleled temperature and rainfall. These results are in keeping with previous studies that report that hoof horn is longer when ground is moist (increasing rainfall) and shorter when ground is hard (increasing temperature).

Whilst our results are only from one farm and in one year, it is clear that climate influences the transmission of FR as reported descriptively in earlier work from Australia (Graham, Egerton, 1968, Depiazzi et al, 1998). Graham and Egerton (1968) stated that *D. nodosus* did not spread when mean daily temperature was <10 °C: in the current study FR spread at mean daily temperatures <10 °C (Fig. 3). It is not clear why these two results appear contradictory, but a laboratory study of survival of *D. nodosus* in small soil microcosms indicated that survival is longer at 5 °C than 15 °C (Cederlof et al., 2013). Other key factors for survival and transmission include moisture level (the farm studied here had high rainfall), soil type and possibly localised evolution of *D. nodosus* strains (Russell et al., 2014).

The incidence rate of FR increased with increasing rainfall in the previous 2 and 4 weeks, probably indicating that moisture is crucial in preventing desiccation of *D. nodosus* and highlighting why the difference between wet and dry climates is pivotal in the probability of elimination of FR from individual flocks (Green and George, 2008). There was no interaction between temperature and rainfall. However these results apply to the UK climate and different relationships with rainfall and temperature might be expected where, for example, warmer weather is associated with higher rainfall.

With regard to routine foot trimming, there were few other causes of lameness than FR in the current study and so it is not possible to determine whether long hoof horn was a precursor to other causes of lameness. However, given the low incidence of other causes of lameness (typical on many farms in the UK (Kaler and Green, 2009)) the effort to maintain all hoof horn just distal to the sole using routine foot trimming would seem, at best, an ineffective use of time. There were sheep on the farm with overgrown horn that would be considered eligible for routine trimming. Approximately 5% of feet had extensive hoof horn overgrowth >1.5 cm (50% longer than normal wall horn length with 1–2 mm distal to the sole) at the start of the study. However, the range of hoof horn lengths was narrower by the end of the study than at the start of the study, possibly because no sheep were lame for long periods of time and so the foot was not diseased and sheep were weight-bearing on all four feet. This suggests that prompt treatment of all lame sheep contributes to self-regulation of hoof horn length and routine foot trimming is not necessary.

## Conclusions

In this study, hoof horn length in ewes at pasture varied through the year; this can be explained in part by temperature, rainfall and FR. New cases of FR were related to the prior higher prevalence of FR and lower prevalence of ID and also prior higher temperature and greater rainfall. Overgrown hoof horn was not a precursor to lameness but occurred once sheep were lame with FR. Once lame, sheep were also at greater risk of low body condition. Treatment of FR led to a return of hoof horn length to that of non-lame sheep whether hoof horn was trimmed immediately or left untrimmed. One year of prompt treatment of all lame sheep led to more uniform hoof horn lengths and on this farm hoof horn length was self-regulating so there was no benefit from routine foot trimming.

## Conflict of interest statement

None of the authors has any financial or personal relationships that could inappropriately influence or bias the content of the paper.

## Appendix A Supplementary material

Supplementary File 1

Supplementary File 2 Maximum hoof length, condition score and age of ewes in the Trim and Inject groups at the start of the study.

## Footnotes

1 See: http://www.martynhicks.co.uk/weather/data.php; last accessed 16 May 2014.
