# Supplementary material for: Dynamics and impact of footrot and climate on hoof horn length in 50 ewes from one farm over a period of 10 months
Source: Vet J. 2014 Sep;201(3):295–301. doi: 10.1016/j.tvjl.2014.05.021 (PMC4168150; doi:10.1016/j.tvjl.2014.05.021)
Supplement: Supplementary File 1 [file mmc1.pdf]

INDIVIDUAL SHEEP CHARACTERISATION SHEET

EID: ____________ SCORER: ____________ DATE: / /

BREED: ____________ BCS: ____________ TEETH *(circle)*: 2 4 6 FM

		LEFT FORE					RIGHT FORE				
		OUTER DIGIT		INNER DIGIT		INTERDIGITAL	OUTER DIGIT		INNER DIGIT		INTERDIGITAL
		Heel & sole	Wall	Heel & sole	Wall		Heel & sole	Wall	Heel & sole	Wall	
Integrity score											
Possible cause											
ID/FR lesion score											
Sticky? *(tick)*											
Overgrowth		TOE	WALL	TOE	WALL		TOE	WALL	TOE	WALL	
Sample	DNA										
	Bact.										

		LEFT REAR					RIGHT REAR				
		OUTER DIGIT		INNER DIGIT		INTERDIGITAL	OUTER DIGIT		INNER DIGIT		INTERDIGITAL
		Heel & sole	Wall	Heel & sole	Wall		Heel & sole	Wall	Heel & sole	Wall	
Integrity score											
Possible cause											
ID/FR lesion score											
Sticky? *(tick)*											
Overgrowth		TOE	WALL	TOE	WALL		TOE	WALL	TOE	WALL	
Sample	DNA										
	Bact.										

Possible cause of damaged/misshapen feet: Not known [**NK**]; Shelly hoof [**SHEL**]; White line disease [**WL**]; Toe abscess [**TA**]; Granuloma [**GR**]; Damaged/misshapen by trimming [**TR**]; Old footrot lesion [**OFR**]; Old scald lesion [**OSC**]; Interdigital growth [**IG**]; Damaged/misshapen by external object [**EX**].

Any additional notes:
