# Supplementary material for: Dynamics and impact of footrot and climate on hoof horn length in 50 ewes from one farm over a period of 10 months
Source: Vet J. 2014 Sep;201(3):295–301. doi: 10.1016/j.tvjl.2014.05.021 (PMC4168150; doi:10.1016/j.tvjl.2014.05.021)
Supplement: Supplementary File 2 — Maximum hoof length, condition score and age of ewes in the Trim and Inject groups at the start of the study. [file mmc2.docx]

**Supplementary File 2**. Maximum hoof length, condition score and age of ewes in the Trim and Inject groups at the start of the study.

|  |  | Inject group | Trim group |
| --- | --- | --- | --- |
| Maximum hoof horn length per foot proud of sole (cm) | Mean | 0.8 | 0.9 |
|  | Median | 1.0 | 1.0 |
|  | Interquartile range | 0.5 – 1.0 | 0.5 – 1.0 |
| Body condition score | Median | 3.5 | 3.5 |
|  | Interquartile range | 3 – 4 | 3 – 4 |
| Number of sheep with the given number of adult incisors | 4 | 0 | 1 |
|  | 6 | 7 | 2 |
|  | Full mouth | 18 | 22 |
