# Supplementary figures and images for: Dynamics and impact of footrot and climate on hoof horn length in 50 ewes from one farm over a period of 10 months
Source: Vet J. 2014 Sep;201(3):295–301. doi: 10.1016/j.tvjl.2014.05.021 (PMC4168150; doi:10.1016/j.tvjl.2014.05.021)

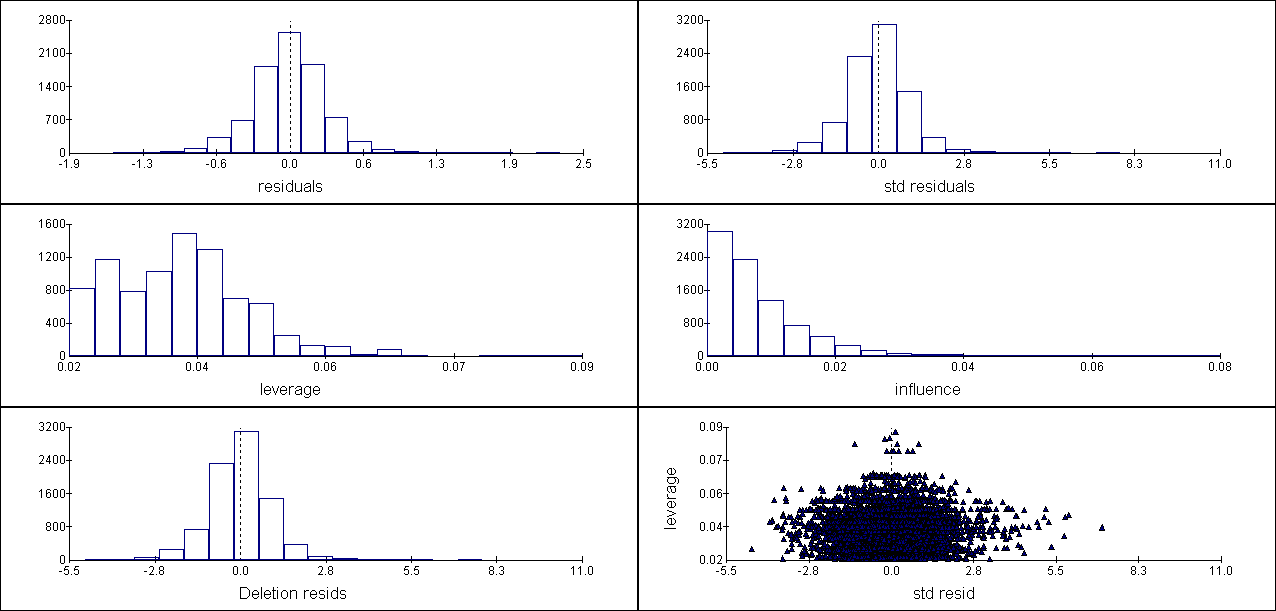

Supplement: Supplementary File 3 [file mmc3.zip › mmc3.tif]
